# Supplementary material for: Patterns and determinants of modern contraceptive discontinuation among women of reproductive age: Analysis of Kenya Demographic Health Surveys, 2003–2014
Source: PLoS One. 2020 Nov 5;15(11):e0241605. doi: 10.1371/journal.pone.0241605 (PMC7643986; doi:10.1371/journal.pone.0241605)
Supplement: S1 Table — (PDF) [file pone.0241605.s001.pdf]

Supplementary table 1: Survival analysis of determinants of contraceptive discontinuation while in need in Kenya 2003 and 2014

Year	2003		2014	
	**Crude HR**	**Adjusted HR**	**Crude HR**	**Adjusted HR**
**Contraceptive method**				
Pill	5.59(2.28-13.70)*	4.11(1.66-10.19)*	7.97(3.77-16.84)*	7.41(3.50-15.70)*
Injectable	2.79(1.15-6.81)*	2.01(0.81-4.96)	5.13(2.45-10.74)*	4.42(2.08-9.38)*
Implant	1.78(0.54-5.84)	1.61(0.50-5.25)	1.80(0.76-4.23)	1.62(0.67-3.90)
Male Condom	6.53(2.70-15.82)*	3.96(1.52-10.28)*	5.01(2.22-11.32)*	3.80(1.70-8.50)*
IUD	Ref	Ref	Ref	Ref
**Age category (years)**				
15-19	3.16(2.25-4.42)*	3.36(2.12-5.34)*	1.50(0.96-2.34)	1.43(0.82-2.51)
20-24	2.37(1.81-3.10)*	2.68(1.82-3.94)***	1.91(1.50-2.45)*	1.65(1.16-2.33)*
25-34	1.53(1.19-1.98)*	1.68(1.22-2.32)*	1.37(1.11-1.70)*	1.21(0.93-1.57)
35-49	Ref	Ref	Ref	Ref
**Residence**				
Urban	0.92(0.77-1.11)	0.93(0.69-1.26)	1.30(1.07-1.57)*	1.31(1.09-1.57)*
Rural	Ref	Ref	Ref	Ref
**Education**				
No education	1.40(0.89-2.21)	1.38(0.82-2.31)	1.21(0.75-1.94)	1.36(0.77-2.38)
Primary	1.42(1.07-1.89)*	1.17(0.84-1.63)	1.11(0.82-1.51)	1.16(0.80-1.67)
Secondary	1.05(0.77-1.44)	0.94(0.66-1.32)	1.08(0.77-1.51)	1.00(0.68-1.46)
Higher	Ref	Ref	Ref	Ref
**Marital status**				
Never married	1.58(1.27-1.97)*	1.30(1.00-1.69)	0.93(0.68-1.28)	0.79(0.56-1.12)
Married	Ref	Ref	Ref	Ref
Single*	0.94(0.69-1.30)	0.96(0.70-1.32)	0.89(0.69-1.15)	0.92(0.71-1.20)
**Religion**				
Catholic	0.86(0.46-1.60)	0.89(0.47-1.68)	0.50(0.26-0.99)*	0.48(0.24-0.98)*
Protestant	0.85(0.46-1.56)	0.91(0.49-1.68)	0.58(0.30-1.13)	0.55(0.28-1.10)
Muslim	0.71(0.35-1.44)	0.77(0.37-1.58)	0.74(0.36-1.54)	0.72(0.33-1.54)
No religion	Ref	Ref	Ref	Ref
Other	1.01(0.35-2.91)	0.96(0.32-2.88)	0.93(0.17-5.22)	0.75(0.13-4.33)
**Number of living children**				
None	Ref	Ref	Ref	Ref
1-2	1.10(0.83-1.47)	1.76(1.23-2.51)*	0.96(0.64-1.44)	0.94(0.58-1.52)
3-4	0.77(0.57-1.05)	1.83(1.19-2.81)	0.76(0.50-1.15)	0.82(0.47-1.41)
5+	0.74(0.53-1.03)	2.43(1.41-4.18)*	0.67(0.43-1.05)	0.86(0.47-1.59)
**Wealth quintile**				
Poorest	1.13(0.83-1.53)	1.01(0.66-1.54)	0.81(0.61-1.09)	1.01(0.71-1.43)
Poorer	1.06(0.84-1.35)	0.95(0.66-1.37)	0.97(0.73-1.28)	1.18(0.86-1.61)
Middle	1.06(0.85-1.33)	0.95(0.67-1.35)	0.95(0.73-1.24)	1.16(0.85-1.57)
Richer	0.99(0.80-1.23)	0.91(0.66-1.26)	0.97(0.75-1.25)	1.11(0.85-1.46)

Richest	Ref	Ref	Ref	Ref
**Fertility Intention**				
Have another	1.28(1.08-1.52)*	1.02(0.83-1.25)	1.29(1.09-1.53)*	1.04(0.84-1.30)
Undecided	1.01(0.60-1.70)	0.88(0.52-1.50)	0.81(0.50-1.32)	0.77(0.47-1.24)
No more	Ref	Ref	Ref	Ref

*Single refers to Divorced/Separated/Widowed
