# Supplementary material for: Patterns and determinants of modern contraceptive discontinuation among women of reproductive age: Analysis of Kenya Demographic Health Surveys, 2003–2014
Source: PLoS One. 2020 Nov 5;15(11):e0241605. doi: 10.1371/journal.pone.0241605 (PMC7643986; doi:10.1371/journal.pone.0241605)
Supplement: S2 Table — (PDF) [file pone.0241605.s002.pdf]

Supplementary Table 2: Profile of ever users of family planning methods											
	**FP Methods**	**LARC methods**					**Short-term methods**				
	**Year**	**2003**		**2014**			**2003**		**2014**		
		**n**	**%**	**n**	**%**	p-value	**n**	**%**	**n**	**%**	p-value
Age categories (Years)	15-19	2	1.6	25	2.9	0.4156	80	4.8	140	3.3	**0.0061**
	20-24	10	8.8	117	13.5	0.1520	408	24.6	937	21.9	**0.0256**
	25-34	57	48.0	458	53.0	0.3059	853	51.3	2320	54.1	0.0522
	35-49	50	41.6	264	30.6	**0.0158**	320	19.3	888	20.7	0.2286
Marital status	Never Married	6	4.9	44	5.1	0.9258	122	7.4	300	7.0	0.5903
	Married	104	87.7	710	82.2	0.1355	1366	82.2	3520	82.1	0.9280
	Single*	9	7.4	110	12.7	0.0960	173	10.4	465	10.9	0.5767
Residence	Urban	56	47.2	432	50.0	0.5668	492	29.6	1883	43.9	**<0.0001**
	Rural	63	52.8	432	50.0	0.5668	1169	70.4	2402	56.1	**<0.0001**
Education level	None	2	1.5	32	3.7	0.2166	60	3.6	110	2.6	**0.0386**
	Primary	33	27.7	454	52.6	**<0.0001**	1050	63.2	2435	56.8	**<0.0001**
	Secondary	53	44.5	238	27.5	**0.0001**	452	27.2	1350	31.5	**0.0012**
	Tertiary	31	26.4	140	16.3	**0.0066**	99	5.9	390	9.1	**0.0001**
Number of living children	None	5	3.8	12	1.3	**0.0417**	53	3.2	174	4.1	0.1055
	1-2	56	46.7	409	47.3	0.9022	777	46.8	2134	49.8	**0.0379**
	3-4	39	33.1	286	33.1	1.0000	556	33.5	1315	30.7	**0.0370**
	5+	19	16.4	157	18.3	0.6135	275	16.5	662	15.4	0.2956
Wealth Quintile	Poorest	2	1.8	74	8.5	**0.0101**	138	8.3	414	9.7	0.0955
	Poorer	6	4.7	132	15.3	**0.0018**	263	15.9	832	19.4	**0.0018**
	Middle	15	12.6	141	16.3	0.3002	341	20.5	913	21.3	0.4974
	Richer	29	24.3	222	25.7	0.7426	392	23.6	1020	23.8	0.8708
	Richest	67	56.6	295	34.2	**<0.0001**	527	31.7	1106	25.8	**<0.0001**
Fertility intention	Have another	42	34.9	347	48.9	**0.0041**	751	45.2	2089	48.9	**0.0104**
	Undecided	5	4.3	34	2.8	0.3671	45	2.7	120	2.8	0.8332
	No more	72	60.8	483	48.5	**0.0119**	863	52.1	2068	48.4	**0.0105**
Religion	Catholic	21	17.7	184	21.3	0.3649	405	24.4	815	19.0	**<0.0001**
	Protestant	91	76.0	634	73.4	0.5458	1168	70.4	3276	76.5	**<0.0001**
	Muslim	5	4.5	31	3.6	0.6262	65	3.9	127	3.0	0.0792
	No religion	2	1.8	15	1.7	0.9372	14	0.9	51	1.2	0.3232
	Other	0	0.0	0	0.0	na	9	0.5	16	0.4	0.5961

*Single refers to Divorced/Separated/Widowed   na=not applicable
