# Supplementary material for: Patterns and determinants of modern contraceptive discontinuation among women of reproductive age: Analysis of Kenya Demographic Health Surveys, 2003–2014
Source: PLoS One. 2020 Nov 5;15(11):e0241605. doi: 10.1371/journal.pone.0241605 (PMC7643986; doi:10.1371/journal.pone.0241605)
Supplement: Attachment — Submitted filename: Comments on PONE-D-20-19927.docx [file pone.0241605.s003.docx]

Comments on PONE-D-20-19927

This paper addresses an important topic. It is well-written, nicely framed, and uses appropriate analytical methods. It was a pleasure to read. I have two primary comments that I raise for the authors’ consideration in their treatment of the discussion/conclusion and abstract, followed by several very minor comments.

Recommendations to prioritize short-term method users and young women seem to come from the finding that these users discontinue at higher rates than users of other methods/older women. However, discontinuation is not necessarily bad or a signal that something in wrong. Just discontinue while still in need (as the authors themselves point out in the introduction). There may be a selection effect: young women choosing condoms because it is easy to discontinue these methods when needs change. Focusing on these women/long-term methods writ large could actually risk ignoring their needs and impairing informed choice. This is more nuanced than is expressed in the abstract (the discussion does a slightly better job of this). I like the focus on resupply of short-term methods.

Similarly, the recommendation to focus on counseling because of the increasing share of reasons for discontinuation being side effects. 1. Did the authors analyze whether the relative increase in side effects as a reason is accompanied or not by an absolute increase in this reason? I.e. are more women discontinuing due to side effects? (Same Q for wanting a more effective method). I’m wary of responding to concerns of side effects with better counseling about side effects. There is a large literature (more developed in high-income countries, but existing globally) about the medical establishment discounting women’s reports of pain and other symptoms. There is also a literature about women’s experiences with side effects. The DHS measure “side effects/health concerns” cannot distinguish whether it is women’s experiences or fears of side effects. We should be careful not to assume that it’s all fears in women’s heads. How do the recommendations to enhance counseling, particularly on side effects, respond to women’s needs if it is women’s experiences of side effects prompting discontinuation? What other recommendations might we consider to ensure that women who experience side effects with their selected method nonetheless have their contraceptive needs met? The issues of counselling and side effects should be a little more developed in the discussion and conclusions section.

Minor comments:

Data are available through The DHS Program website. MEASURE DHS was an old contract and references to that name should be updated. Likewise, ICF International is now ICF.

Proofread: In at least one instance, the 2008-09 Kenya DHS appears as “2008/8” (p2, line 27).

Do you want more consistency between the terms “family planning” and “contraception” (or modern contraception) or is this distinction intentional?

Is uptake distinguishable from use? I believe, when discussing prevalence, use may be the more accurate term (p5, line 89).

P6, line 122: Do you mean “adoption” instead of “application”?

P7, line 126: It would more consistent to use “episodes” in lieu of “incidents”.

Can Table 1 be formatted to have some separation between or better alignment between the N and (%)? It is a little difficult to read as is.

When discussing changes in 12-month discontinuation rates across surveys (p10), it may be worth noting any substantial shifts in the method mix over that time period.

Please use aHR consistently. On p11, lines 199-200 refer to the aHR, but have it labeled HR, making it unclear whether it is the crude or adjusted hazard rate, without referring to the table. (Alternately, the crude HRs could be removed from Table 3.)

In Table 4 (p13), the na* on the row for “up to God/fatalistic” is in bold instead of plain text.

Presentation of data on reasons for discontinuation (p13) exclude all fertility/need-based reasons. I assume that these reasons make up the difference between the sum of the reasons presented in Table 4 and 100%, i.e. that the authors are presenting the relative share of each reason to all reasons for discontinuation. This should be clarified, particularly if I am wrong in this assumption.

In the discussion (p16), the authors summarize their results in terms of likelihoods. While there is a correspondence between likelihoods and hazards or hazard ratios, technically, the authors calculated the latter, not the former. I’m not sure this is a point worth getting worked up about, though.
