# Supplementary material for: Patterns and determinants of modern contraceptive discontinuation among women of reproductive age: Analysis of Kenya Demographic Health Surveys, 2003–2014
Source: PLoS One. 2020 Nov 5;15(11):e0241605. doi: 10.1371/journal.pone.0241605 (PMC7643986; doi:10.1371/journal.pone.0241605)
Supplement: Attachment — Submitted filename: Response to Reviewers.docx [file pone.0241605.s005.docx]

**Re: Revision of Manuscript “Patterns and determinants of modern contraceptive discontinuation among women of reproductive age: Analysis of Kenya Demographic Health Surveys, 2003–2014”**

**Comments**

1. **On page 5, line 105, the authors refer to the method mix, but it appears that everything in lines 101-108 is prevalence of each method (the percentage of women using each method) rather than method mix (the percentage of use contributed by a particular method).**

**Response:** *This is noted. We have since updated the figures from line 98-106 and we are now reporting contraceptive method mix.*

2. **Optional: Also in this section, the authors not trends that are both substantial and likely statistically significant (e.g. increase of injectable use from 14.3% to 21.6% in 2003-2008/9) and those that are not (e.g. decrease of pill use from 7.5% to 7.2%). If the authors would like to streamline the paper, this is one paragraph where some modest edits could help achieve that.**

**Response:** *We take note of this comment. Ascertaining whether the changes observed in the trends of the method mix are statistically significant, is beyond the scope of our paper. We have however revised the write up to report on the changes observed without using the terms increase or decrease.*

3. **On page 7, lines 149-150, the authors are technically correct that discontinuation while in need comprises women who discontinue while still at risk of becoming pregnant, do not want to become pregnant… However, isn’t this definition operationalized from the reasons for discontinuation (given in the second column of the calendar)? It would be helpful to clarify this and list which reasons comprise discontinuation while in need (e.g. husband disapproval, side effects, access, cost, wanted more effective method…) and those that do not comprise while in need (wanted to become pregnant, husband away/infrequent sex,…).**

**Response:** *This is noted. We have since updated the write up to reflect the suggestion in lines 148-153*

4. **Thank you for clarifying that percentages in Table 4 do not sum to 100% because the table excludes discontinuation due to no further need. Could the table titled be revised to read, “Reasons for discontinuation while still in need” rather than “…among women in need,” as this change would be more accurate? And could the authors add a footnote to the tables similar to their response to the reviewer, e.g. “Percentages do not sum to 100% since women who discontinued while not in need of contraceptives, including those who reported they wanted to become pregnant/experienced menopause (2003, 42.1% and in 2014, 46.5%) were excluded from the analysis.”**

**Response:** *We have revised the title of the table as advised. We have also added a footnote.*
